# The mechanical test as a supplemental decision support tool for the safe removal of an Ilizarov circular external fixator

**DOI:** 10.1186/s40001-024-02258-9

**Published:** 2025-02-07

**Authors:** Yulin Xu, Jialin Liu, Jinghong Yang, Tao Zhang, Zhong Li, Yanshi Liu

**Affiliations:** 1 Department of Orthopaedics, the Affiliated Hospital of Southwest Medical University, Luzhou, Sichuan China; 2 Department of Oral Implantology, the Affiliated Stomatology Hospital of Southwest Medical University, Luzhou, Sichuan China; 3 Department of Orthopedics and Trauma, Tianjin Hospital, Tianjin, China

**Keywords:** Axial load–share ratio, Bone healing, Ilizarov external fixator, Timing of fixator removal

## Abstract

**Background:**

Timing the fixator removal is vital for a successful external fixation treatment. The purpose of this study was to determine the effectiveness of axial load–share ratio in vivo as a supplemental decision support tool for the safe removal of an Ilizarov circular external fixator.

**Methods:**

This prospective observational study consists of 83 patients undergoing tibial or femoral lengthening with Ilizarov circular external fixation in our institution, from January 2011 to October 2019. In group I (38 patients), the external fixator was removed based on the surgeon’s clinical experience and radiographs from January 2011 to June 2015. In group II (45 patients), from July 2015 to October 2019, the supplemental axial load–share (LS) ratio test was accomplished without the knowledge of the clinical results by another medical team. The test was performed by electronically measuring forces in the fixator rods and in a ground force plate. When the LS ratio < 10% was consistent with the conclusion (dense bone formation was achieved in the distraction zone) drawn from the corresponding routine radiographs by the treating surgeon, the external fixator was removed.

**Results:**

There was no statistical significance in demographic data between the two groups (*P* > 0.05). In group I, 4 of the 38 patients suffered refracture (the refracture rate was 10.5%) after fixator removal, and bone union was finally achieved with further intervention by intramedullary nail. In group II, 36 patients terminated the external fixation after the first mechanical test, and another 9 patients terminated the external fixation at the subsequent test. None of the 45 patients in group II suffered refracture (the refracture rate was 0%). There was statistical significance in the refracture rate between the two groups (*P* < 0.05).

**Conclusions:**

Adequate assessment of bone regenerate is crucial before removing an external fixator to prevent deformation or refracture. The axial load–share ratio in vivo is a practically quantitative method to supplement radiography and clinical experience for the assessment of regenerate healing, and the axial load–share ratio dropped below 10% is a safe limit for the Ilizarov circular external fixator removal.

## Background

The treatment of massive bone defects, infected nonunion, limb deformity, and high-energy fractures with significant soft tissue damage, where internal fixation is impossible or inadvisable is a challenge for orthopedic surgeons. The external fixator has played an important role in managing these complex problems [1–5]. During this treatment, information about the healing process is necessary to adjust patient's load-bearing capacity and the time for removing the external fixator.

Most patients wish to remove the external fixator as early as possible due to the inconvenience of wearing the device. Early removal of the external fixator introduces the risk of deformation or refracture, but infection or limitation of joint motion is increased if removal is delayed. Therefore, the most critical decision in the process of external fixation is when to remove the external fixator [6]. Traditional assessment of bone healing is usually performed by radiographs in two planes, and strongly depends on the surgeon’s clinical experience [7] which is an imprecise guide [8]. In addition, these radiographs only allow qualitative assessment of new bone formation and lack quantitative information, thus providing limited guidance for the decision of fixator removal [9]. An objective, quantitative and easy method for monitoring bone healing is needed to prevent unnecessary long treatments or incorrect timing of the fixator removal.

Quantitative methods, such as dual-energy X-ray absorptiometry (DEXA) [10, 11] and quantitative computed tomography (QCT) [12], have been proposed to predict load-bearing capacity non-invasively. However, these techniques may involve large radiation doses. The high correlation between the stiffness of the bone regenerate and its strength contributed to stiffness measurements in the quantitative evaluation of load-bearing capacity [13]. Aarnes et al. designed a system for in vivo testing of axial stiffness in regenerate tissue, concluding that the external fixator can be removed safely when the load–share (LS) ratio dropped below 10% [14]. The theoretical basis of this method is that an externally applied load is shared between the fixator and the regenerating bone; the amount of load carried by the regenerate depends on its axial stiffness which increases with advanced mineralization. By measuring the force in the fixator while applying a known external load to the limb, the load–share ratio between fixator and limb can be assessed.

Therefore, the purpose of this article was to evaluate the effectiveness of axial load–share ratio in vivo as a supplemental decision support tool for the safe removal of Ilizarov circular external fixator in our clinical cases.

## Methods

First of all, we retrospectively collected a group of 38 patients (group I) undergoing tibial or femoral lengthening with Ilizarov external fixation in our institution, from January 2011 to June 2015, including 31 males and 7 females with a mean age of 38 years (range 19–63 years). The external fixator was removed depending on the traditionally radiological and clinical assessment by the treating surgeon (dense bone formation was achieved in the distraction zone).

Starting in July 2015, we focused on a new assessment method (axial load–share ratio in vivo) of the strength of the regenerate bone and ultimately to assist in the determination of when it is appropriate to remove the frame. 45 patients (group II) undergoing tibial or femoral lengthening with Ilizarov circular external fixation in our institution were prospectively collected, from July 2015 to October 2019, including 40 males and 5 females with a mean age of 41 years (range 21–62 years). In this group, according to the mathematical analysis and clinical conclusion of Aarnes et al. [14], the external fixator was removed when the conclusion (LS ratio < 10%) that drawn from the mechanical test by another medical team is consistent with the radiographs and clinical assessment by the treating surgeon.

All the 83 patients were treated by the same team. Patients with poor compliance, age > 65 years, and any other illness that can affect bone healing (such as diabetes, hypertension, osteoporosis, kidney disease, etc.) were excluded. Informed consent was obtained from all patients for their data to be recorded and published in our study. The Ethical Committee of our institution approved this study.

### Theory of mechanical test

According to Aarnes et al. [14], the amount of load carried by the regenerate is proportional to its stiffness. The load–share ratio, which is assessed by the external load and the force in the fixator, is defined as the compressive force in the fixator rods divided by the applied external load. The external load is transferred completely through the fixator when the regenerate stiffness is zero, and the LS is 100%. Subsequently, the LS decreases as the regenerate gradually stiffens due to a larger amount of the load is carried by the new bone. The load–share ratio, therefore, is an indirect and objective index about the load-bearing capacity of the regenerate.

**Fig. 1:**
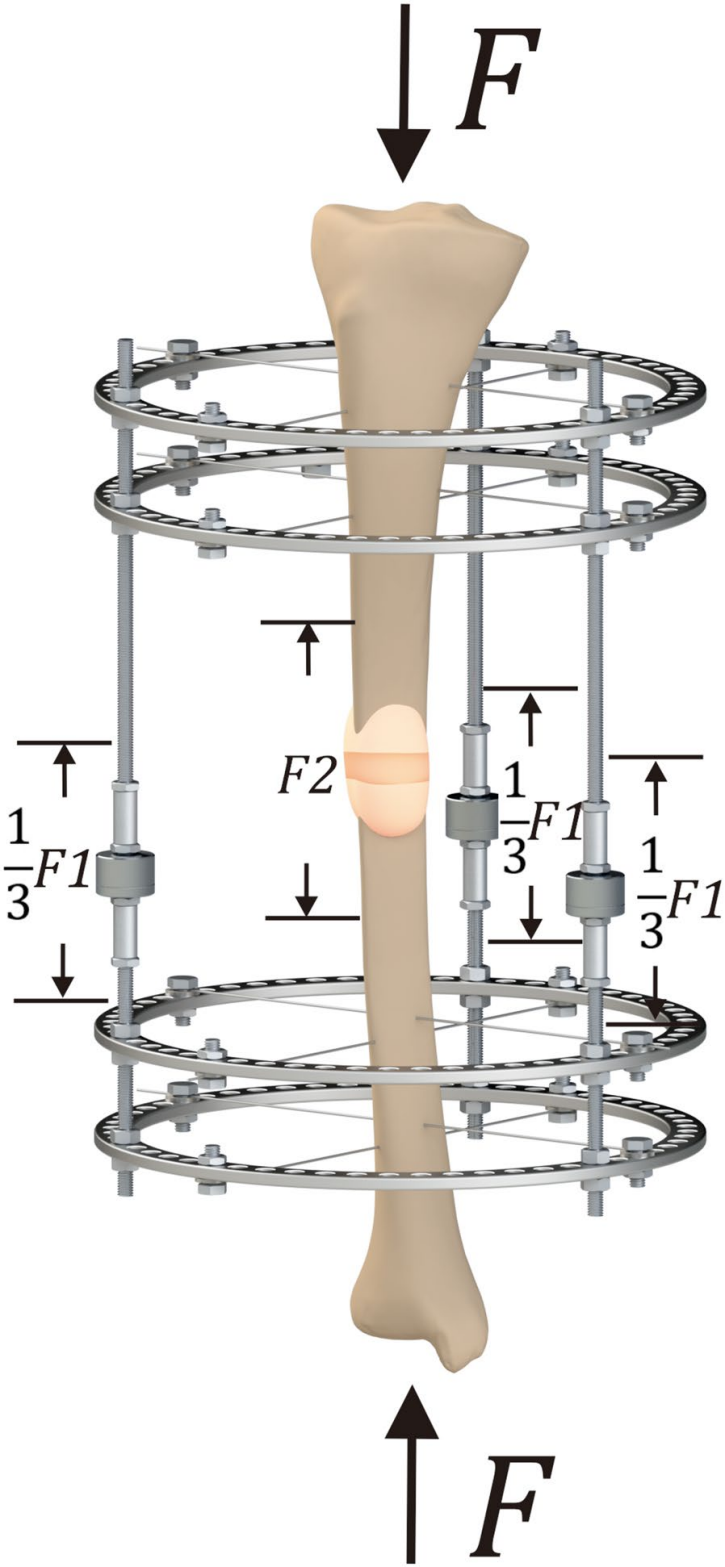
Three-dimensional diagram of the injured limb and external fixator with force sensors. *F* is the total force applied externally on the injured limb. *F1* is the force shared between the fixator rods, and *F2* is the load carried by the regenerate

The externally applied load is defined as *F* in the present study, shared between the fixator (*F1*) and the regenerate (*F2*), respectively. The load carried by the fixator is obtained by adding the carried load of each fixator rod measured by the force sensors. In this simplified model, *F1* is shared equally between the rods based on the assumption that the system is symmetric both vertically and horizontally (Fig. 1).

Therefore, the definition of load–share is

1 $$LS = \frac{F1}{F}$$

### Device for force measurement

**Fig. 2:**
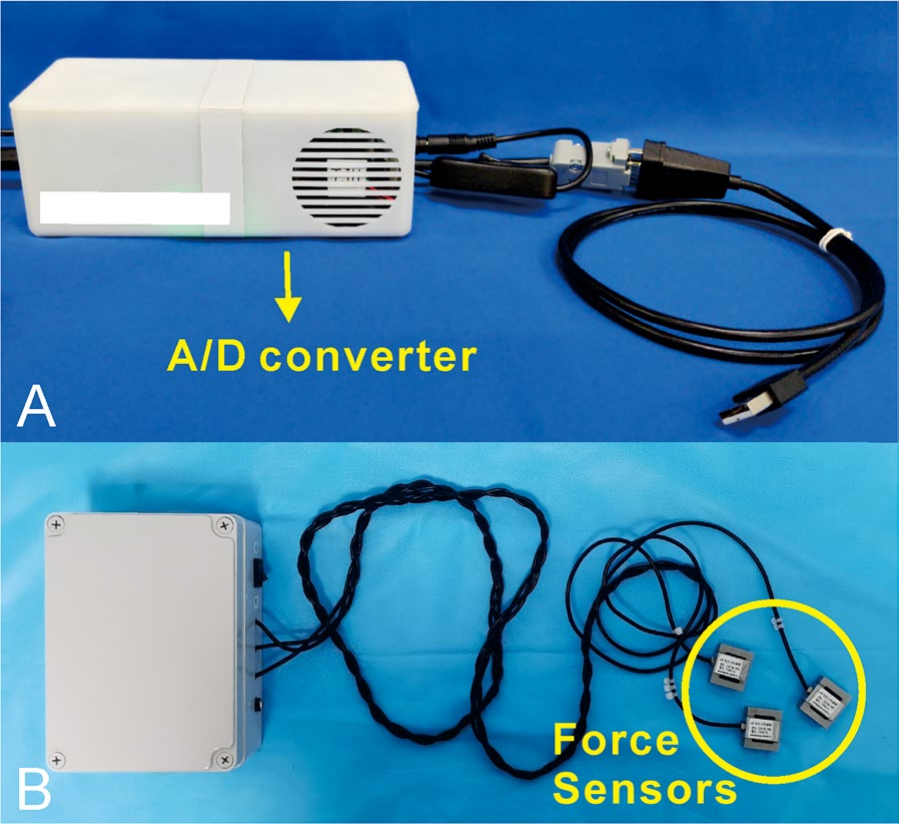
Force collecting devices. **a** Custom-made A/D converter. **b** Three dismountable force sensors for measuring the force carried by the fixator

The complete device to measure forces consisted of three dismountable force sensors (maximum load of 1000N, HYLY-019, Bengbu Hengyuan Sensor Technology Co., China) on the fixator rods, a custom-made A/D converter, a force platform (maximum load of 1200N, RGZ-120, Jiangsu Suhong Medical Instrument Co., China), and a customized computer software. The force sensor on each fixator bar is used to measure the force carried by the fixator; the output signals are processed and wirelessly transmitted to the computer software through the A/D converter. The external load is equal to the ground reaction force and measured by the force platform. The customized computer software performs the analyses and records the data (Fig. 2).

**Fig. 3:**
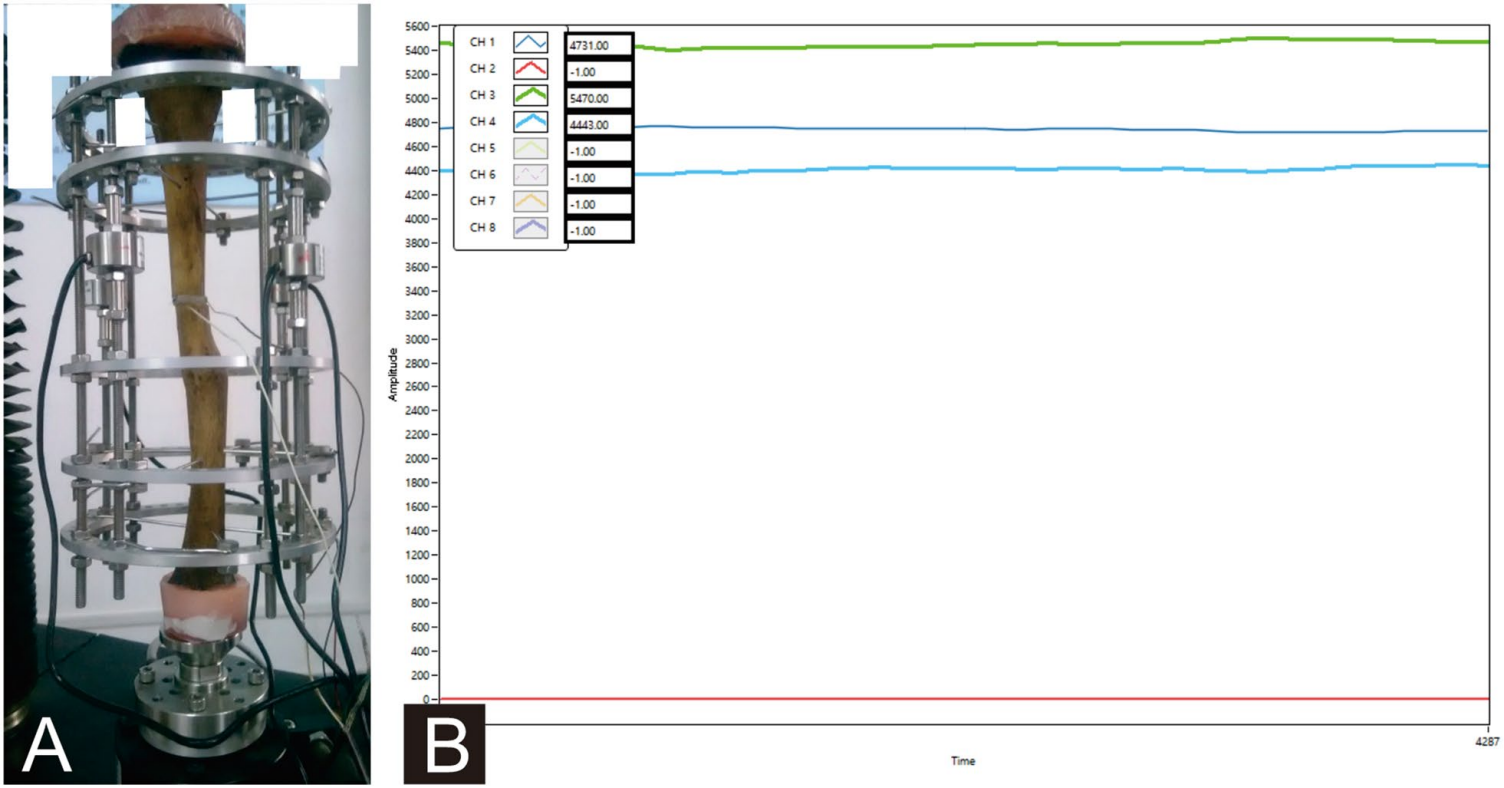
**a** Effectiveness of the complete device evaluated by a material test machine. **b** Representation of the custom-made computer software

A material test machine (BOSE Electroforce 3150, USA) was used to calibrate the force sensors and evaluate the effectiveness of the complete device (Fig. 3).

### Clinical application

The external fixator was planned to be removed based on the treating surgeon’s clinical experience and radiographs in which sufficient consolidation of the distraction zone (dense bone formation) was achieved. Simultaneously, the axial load–share ratio test was accomplished by another medical team without the knowledge of clinical results.

In the test procedures, the force sensors are attached to a separate set of rods and then temporarily connected between the rings in the fixator. Three bars with sensors are sufficient to keep the system stable; the original rods are still in place, but loosened and bypassed by bars with sensors. The measuring bars took over the fixator load completely during the measurements, including inherent stresses of the bone–soft-tissue-fixator mounting. To measure the relative change in force, the load cells were zeroed before the test, and then the carried load is saved and appeared by the computer software. Both the force platform and the force sensors are sensitive to axial force only, and the load–share is thereby the ratio between axial load in the fixator and the regenerate. The evaluations were performed with the Excel program (Microsoft). The original rods were reattached, and rods with the embedded sensors were removed after the mechanical test.

Static test was performed during the procedures. The test was conducted by loading the limb with a known force (full weight bearing or external compressive force within 300N, according to our usual clinical practice), and the investigator must take care that the patient was relaxed during the procedure to minimize the effect of muscle activity (Fig. 2b). For an accurate measurement, the static test was conducted three times to obtain the mean valid forces.

When the conclusion drew from the mechanical test (LS ratio < 10%) is consistent with the decisions of the treating surgeons made on the basis of the corresponding routine radiographs (dense bone formation was achieved), the external fixator was dynamized involving gradual loosening on rods and weight-bearing on loose rods and then be removed. If not, continuing treatment in the fixator and a mechanical test was performed every 2 weeks.

All patients were put on the functional brace for 4–6 weeks to prevent refracture after fixator removal. They were warned to use the injured limb only as much as necessary and report any adverse events or symptoms. Furthermore, review and radiographs 2 weeks later after frame removal were routinely conducted. All patients were followed up for at least 12 months.

### Statistical analysis

Statistical analysis was performed with the SPSS 22.0(IBM Corp, USA). Data were evaluated by normality test first. Continuous variables were expressed as the mean and range, and analyzed by Independent-samples *T* tests or Mann–Whitney *U* test. The count variables were analyzed by the Chi-square or Fisher’s test, expressing as number. Statistical significance was set at *P* < 0.05.

## Results

The complete device was capable of measuring the axial load–share. The demographic data of the two groups are shown in Table 1, and there was no statistical significance (*P* > 0.05). However, for the refracture rate, a statistically significant difference was observed between the two groups (*P* < 0.05).

**Table 1:** Details of patients in the two groups

	Group I	Group II	Statistical value	*P* value
Mean age in years	38 (19–63)	41 (21–62)	−1.171	0.245
Gender (male:female)	31:7	40:5	0.890	0.345
Injured bone (tibia:femur)	33:5	42:3	–	0.460
Mean lengthening size(cm)	5.8(3–12)	5.9 (3–11)	−0.348	0.729
Mean external fixation time (weeks)	37.0 (26–63)	38.8 (25–61)	−0.929	0.355
Mean time of follow up (months)	16.3 (12–25)	17.3 (12–26)	−1.063	0.291
Refracture rate	10.5% (4/38)	0% (0/45)	–	0.040

In group I, the mean lengthening size was 5.8 cm (range 3–12 cm), and the mean external fixation time was 37.0 weeks (range 26–63 weeks). Four patients suffered refracture after frame removal, and the refracture rate was 10.5%. Bone union was finally achieved with further intervention by intramedullary nail.

**Fig. 4:**
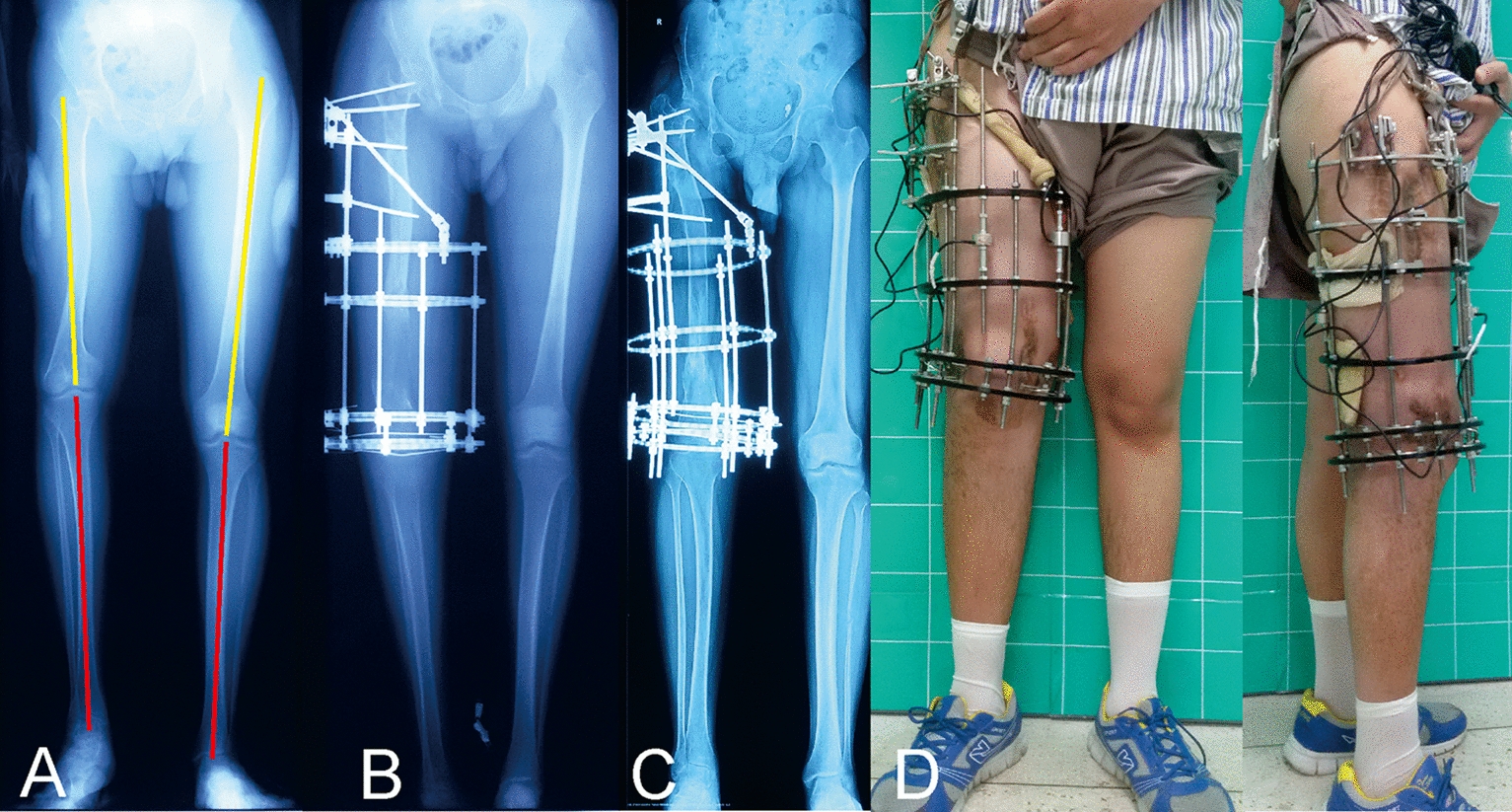
Images of a 20-year-old male patient with limb discrepancy in right femur treated by the circular external fixator using Ilizarov distraction osteogenesis technique. **a** Initial radiograph manifests the limb discrepancy (9 cm) in the right femur. **b** Radiograph immediately after the target length is achieved. **c** Bone lengthening was completed with good regenerate consolidation before the removal of the external fixator. **d** General appearances during the mechanical test, and the axial load–share ratio is 6.5% at that time

**Fig. 5:**
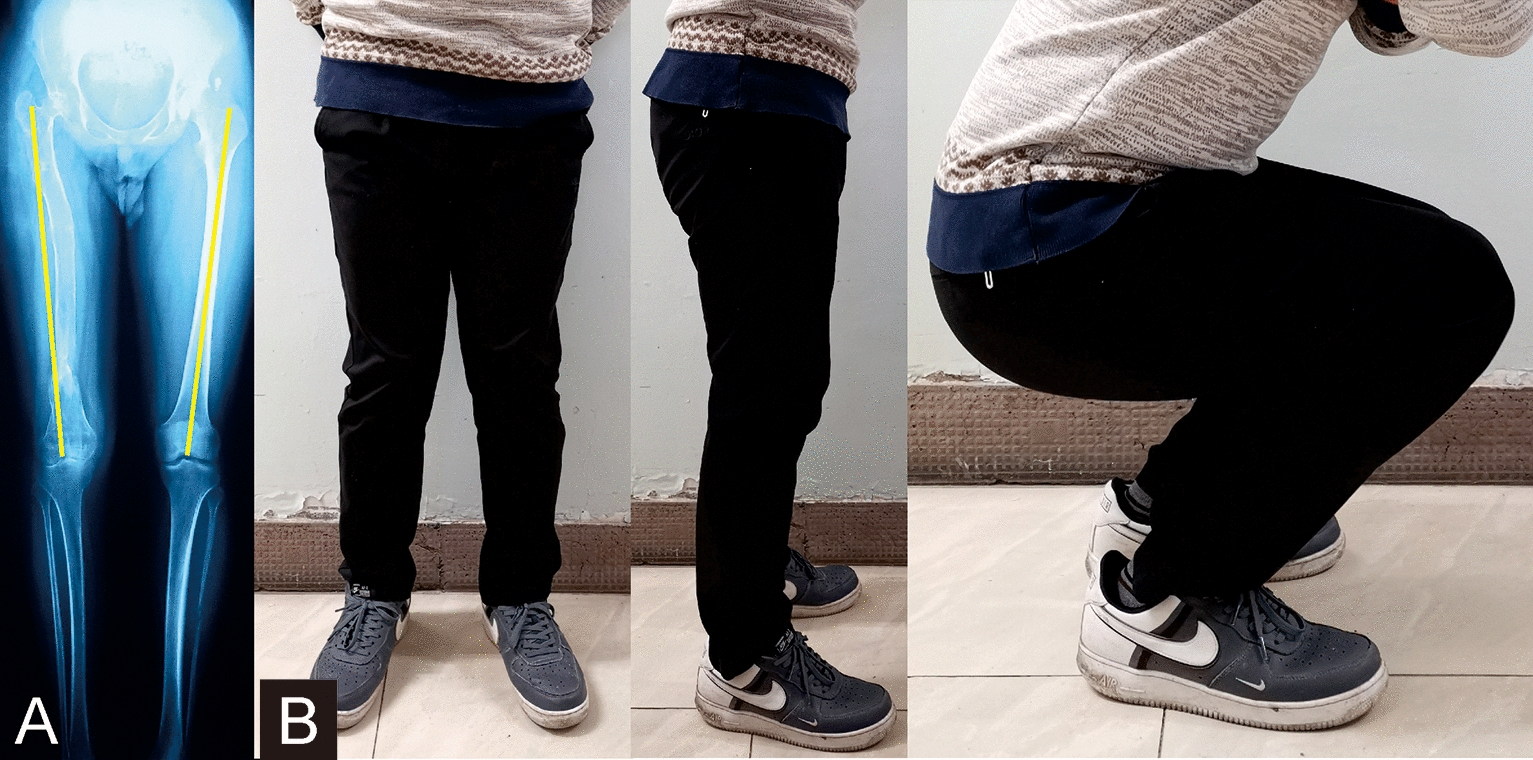
Follow-up images of the same patient (shown in Fig. 4) after removing the external fixator. **a** Radiograph 6 months later. **b** General images at the last visit 13 months later, showing the excellent functional results

In group II, the mean lengthening size was 5.9 cm (range 3–11 cm). None felt any discomfort during the testing procedures. Thirty-six patients showed axial load–share ratio below 10% (range 0.7–9.1%) at the first test and underwent fixator removal. Another 9 patients who showed axial load–share ratio exceed 10% (range 10.5–15.2%) got continuing treatment in the external fixator at the first test (More details are shown in Table 2). After a mean time of 3.6 weeks (range 2–6 weeks), the external fixators were safely removed when the axial load–share ratio below 10% (range 2.6–8.9%). The mean external fixation time of these 9 patients was 33.7 weeks (range 27–39 weeks). The average total external fixation time of the 45 patients was 38.8 weeks (range 25–61 weeks). None of the 45 patients suffered refracture after frame removal. (Typical case of the mechanical test is shown in Figs. 4 and 5).

**Table 2:** Details of the nine patients who underwent two mechanical tests in group II

Case	Gender	Age (year)	Injured bone	First time (W)	First LS ratio (%)	Second time (W)	Second LS ratio (%)
1	Male	41	Tibia	26	15.2	30	8.9
2	Male	20	Tibia	34	11.3	36	6.5
3	Male	57	Tibia	33	12	39	8.6
4	Male	49	Tibia	28	11	32	4
5	Male	28	Tibia	25	10.5	27	2.6
6	Female	52	Tibia	34	13.4	38	6.8
7	Male	45	Tibia	33	14.6	37	7.4
8	Male	36	Tibia	26	11.5	30	6.2
9	Male	39	Femur	33	12.8	35	7

First time: time elapse from initial external fixation to the first mechanical test

Second time: time elapse from initial external fixation to the second mechanical test

*LS ratio* load–share ratio

## Discussion

Bone regenerate is a complex process, which has been widely studied through experimental techniques [15, 16]. However, how to monitor bone regenerate in humans is still a challenge. Monitoring of bone healing is routinely done by clinical evaluation and radiographic examinations, but it strongly depends on the clinical experience and lacks the quantitative information about callus strength that would be helpful for therapeutic decisions [6].

Of all the methods for achieving long bone healing, one alternative is to use an external fixator to restore the original stiffness and the mechanical stability of the bone. The external fixation is mainly responsible for load transfer through the injured bone and creates a suitable mechanical environment for bone regenerate [17]. The device acts as a mechanical bridge, allowing partial recovery of the load transfer through the injured member and decreasing the interfragmentary movement.

Leaving the external fixator for longer than necessary would lead to various complications, such as limitation of joint motion due to contracture. As early as 1983, Terjesen et al. [18] concluded that there was a stress-protecting effect of the fixation frame on the bone, and the external fixation should be removed as soon as the fracture healed to avoid this effect. However, premature removal of the frame also leads to severe complications, including fracture or axial bending at the callus. Ilizarov himself also remarked that “leaving the apparatus on for longer than necessary is as harmful as removing the fixator too early” [19]. Therefore, choosing an appropriate time to remove the external fixator is essential for successful treatment.

Several imaging modalities have been proposed to estimate the status of the regenerative callus tissue, such as high-resolution magnetic resonance [20, 21], quantitative ultrasound [22], dual-energy X-ray absorptiometry (DEXA) [11], and quantitative computed tomography (QCT) [12, 23]. However, these alternative methods may involve large radiation doses, be restricted by cost and availability, or have not been assessed adequately for reliability. Besides, Fischgrund et al. [24] specified the presence of three of the four cortices of a minimum 2 mm thickness as a guideline for removing the fixator, and they presented a re-fracture rate of only 3%, while Starr et al. [25] attributed the good results obtained by Fischgrund et al. [24] to the better clinical judgment of an experienced surgeon involved in decision making, rather than the radiographic criteria demonstrated in their study. Hazra et al. [26] made a retrospective study of 70 patients to compare the BMD ratio and pixel value ratio, concluding that pixel value ratio is a good method for assessing callus stiffness as well as judge the timing of fixator removal, while the inherent limitation is that the pixel value is easily affected by the presence of metal in the vicinity of the point of measurement. Briefly, none of the aforementioned methods has acquired gold standard status.

Resistance to deformation is a fundamental property of a structure and is defined as its stiffness, which seems to be an appropriate measure of bone regenerate. As Goodship et al. [27] showed, there was an increase in stiffness and stability of regenerated bone after fracture healing during time progression. Information on the rate of increase of the mechanical properties of a healing bone is, therefore, valuable in determining both the rate at which a fracture will heal and in helping to define an objective and measurable endpoint of healing. As early as 1972, Jorgensen [28] described a mechanical method of measuring the bone deflections during load bending to measure the deflection on Hoffmann-treated crural fractures. Subsequently, clinical in vivo applications of mechanical measurements in fracture healing have been published.

Richardson et al. [29] measured fracture stiffness in 212 patients with tibial fractures treated by external fixation, considering that stiffness of 15 Nm/degree in the sagittal plane provides a useful definition of the union of tibial fractures. Wade et al. [30] studied the progression of healing in 103 unstable fractures of the tibia, advocating that fracture stiffness should be measured in two orthogonal planes when assessing tibial healing and suggesting that values above 15 Nm/ degree in two planes indicate to remove the fixator safely. These studies are all concentrated on the direct measurement of callus stiffness, which allows a good estimation of the load capacity of the healing bone; however, this method is limited by the removal of the fixator for each measurement. Furthermore, in the early phase of bone healing, it is impossible to remove the fixation device due to the potential risk of losing the reduction under loading. This procedure is thereby only applicable for the later phases.

For clinical applications, however, most often, only the deformation in the longitudinal axis of the bone was measured. Another possibility to measure the load sharing between bone and fixator is the integration of a load cell in the fixator body. Evans et al. [31] developed a transducer that been fitted to the support column of an external fixator to determine the stiffness during the healing process. Seide et al. [32] described a hexapod system that can be used for measuring axial and shear forces as well as torsion and bending moments in the fixator in vivo, concluding that the measured values enabled both the type of fracture to be determined as well as the monitoring of the healing process. Aarnes et al. [14] presented an in vivo test for assessment of regenerate axial stiffness after the distraction phase of lengthening therapy. In their clinical trial of 22 individuals with tibia1 lengthening, the fixator was removed when the load–share ratio dropped below 10%, and none experienced refracture. Therefore, they drew the important conclusion that the external fixator can be removed safely when the load–share ratio dropped below 10%.

Recently, Mora-Macias et al. [33] performed a bone transport experiment in sheep, the forces through the fixator evolution were measured, and the callus stiffness was estimated from these forces. Their data complement previous experimental and computational works. They also concluded that the force and stiffness data together with conventional methods such as radiographs might contribute to know exactly when the limb stiffness is recovered, while the fixator is implanted, or estimate the optimum time when the fixator should be retired.

Refracture after the frame removal was one of the few major complications reported by De Bastiani when the external fixation was used, affecting 3% of patients [34]. Others have reported rates of 6% [35] and 9.4% [36]. In the present study, we conducted the axial load–share test in 45 patients (group II) who underwent Ilizarov circular external fixator treatment in the lower extremity and the evaluation criteria of Aarnes et al. [14] were continuously used. With a mean of 17.3 month follow-up, there was none experienced refracture after removing the external fixator with an axial load–share ratio less than 10%. While in group I, the frame was removed just depending on the traditionally radiological and clinical assessment. 4 of the 38 patients suffered refracture after the frame removal, and the refracture rate was 10.5%. There was statistical significance in the refracture rate between the two groups. The results manifested that the mechanical test as a supplement to radiography for evaluating the regenerate healing made the fixator removal safer.

The regenerate healing is generally defined as the reconstruction of the bony biomechanical characteristic. For bone union assessment, it is traditionally evaluated using imaging modalities that cannot provide related biomechanical information. There were 9 patients that the treating surgeon had decided to remove the frame in group II, but the mechanical test has overruled this decision in this study. After a period of time, the external fixator was safely removed based on the axial load–share ratio dropped below 10%. We, therefore, speculate that it may due to the biomechanical properties of the regenerated bone itself were not completely recovered, but the radiographs provided inaccurate healing information.

Aarnes himself also emphasized that “A small bone bridge may carry a significant load and, therefore, cause a low LS ratio without the bone being completely healed” [14]. Therefore, they suggested that radiographs must be taken to assess the geometry of the new bone. For our experience, the radiographs, load–share tests, and clinical experience complement each other in evaluating regenerate healing. A prudent attitude and comprehensive assessment should be adopted regarding the removal of an external fixator. We also advocate that the LS ratio should be measured in both static and dynamic tests when assessing regenerate healing for more excellent safety.

The axial load–share test provides an objective assessment of bone regenerate, including potential advantages of fewer radiographic images taken (lower cost) and a lower ionizing radiation dose. There is no need to remove the fixator when this indirect and non-invasive method was performed. It is possible to measure the load sharing and indirect callus stiffness even from the first day postoperatively without the likelihood of fracture, malunion, and pseudarthrosis. Furthermore, the total device is price-friendly and manufacture-simply. This technique does not involve complex procedures and electronic devices that remain for a long time or even forever in patients. There are potential chances for its wider use in most fracture clinics, as it supplements radiography and clinical experience and makes us safer while removing the fixator.

The present study had several limitations. First, considering its relatively small sample size in a single center, a prudent attitude should be adopted to interpret the potential greater risk of refracture if the fixator was removed based on clinical assessment only. Furthermore, this method is concentrated on the axial load, because the sensors are sensitive to axial force only; the clinical application thereby may be limited by the spatial structures of the external fixator, such as the hexapod external fixator, which contains multi-directional forces in each rod. In addition, other tests are required to determine whether there is another preferable limit of LS ratio for regenerate healing assessment.

## Conclusion

Adequate assessment of bone regenerate is crucial before removing an external fixator to prevent deformation or refracture. The axial load–share ratio in vivo is a practically quantitative method to supplement radiography and clinical experience for the assessment of regenerate healing, and the axial load–share ratio dropped below 10% is a safe limit for the Ilizarov circular external fixator removal.

## Data Availability

The data sets analysed during the current study are available from the corresponding author on reasonable request.

## Abbreviations

LS: Load–share
DEXA: Dual-energy X-ray absorptiometry
QCT: Quantitative computed tomography
